# Cross-sectional analysis of coding, patient characteristics, consultation frequency and pharmacological treatment of adults with severe mental disorders in Irish general practice

**DOI:** 10.1007/s11845-021-02747-4

**Published:** 2021-09-08

**Authors:** James Larkin, Ivana Pericin, Brian Osborne, Philip Dodd, Claire Collins

**Affiliations:** 1 Irish College of General Practitioners, 4-5 Lincoln Place, Dublin, Ireland; 2 Mental Health Services, Health Services Executive, Dublin, Ireland

**Keywords:** Bipolar disorder, Clinical coding, Depressive disorder, Prescriptions, Schizophrenia, Severe mental disorders

## Abstract

**Background:**

General practitioners are the gatekeepers of Irish healthcare and they offer continuity of care to patients. Irish general practice is therefore considered appropriate for preventing, diagnosing and managing most mental health problems.

**Aims:**

This study sought to establish the coding frequency, consultation frequency, patient characteristics and pharmacological treatment of patients with severe mental disorders (SMDs) in Irish general practice.

**Methods:**

A cross-sectional design was used. A *finder* tool embedded in the practice software assisted general practitioners (GPs) coding adult patients with SMDs. Eleven practices uploaded anonymous data on 2,203 patients. Variables analysed included disease code, consultations, prescriptions, sex, patient status and age.

**Results:**

Overall, 2.9% (*n* = 2,337) of patients had ever been coded with a SMD, 2.4% (*n* = 1,964) coded with depressive disorder ever and 0.26% (*n* = 209) and 0.3% (*n* = 233) with bipolar disorder and schizophrenia, respectively. Overall, 68.0% (*n* = 1,336) of patients with depressive disorder were female, and 74.0% (*n* = 171) of patients with schizophrenia were public patients. The median consultation rate in the previous 3 years was highest for schizophrenia patients at 24.5 visits.

**Conclusions:**

Coding of SMDs in Irish general practice appears incomplete. Patients with SMDs have high consultation rates. Patients with depressive disorder are more likely to be female and public patients. This research suggests that the improvement of coding in Irish general practice is the first practical step required to detecting prevalence rates.

**Supplementary Information:**

The online version contains supplementary material available at 10.1007/s11845-021-02747-4.

## Background

Severe and enduring mental disorders (SMDs), which include bipolar disorder, schizophrenia and recurrent depressive disorder (two or more major depressive episodes in an individual), have many negative consequences for patients [1, 2]. SMDs lead to a high degree of morbidity — 54.9% of disability adjusted life years lost as a result of mental and substance use disorders are accounted for by SMDs [3]. There is also a higher mortality rate for patients with a SMD. The life expectancy of patients with a SMD is shorter than members of the general population [2, 4].

Primary care can be considered an appropriate setting to analyse SMDs and their treatment for several reasons. General practitioners (GPs) constitute the largest group of healthcare professionals in primary care in Ireland. GPs are the gatekeepers of Irish healthcare and they offer continuity of care to patients. With additional investment, it is thought that Irish primary care could be ‘ideally situated’ for preventing, diagnosing and managing most mental health problems [5]. In one survey [6], Irish GPs estimated that 95% of patients with mental health issues received their care in primary care. Mental health policy in Ireland [7, 8] considers primary care as pivotal in the care of patients with mental illness.

Clinical disease coding systems in general practice offer a means of tracking illnesses amongst large populations of patients, as well as consultations and relevant prescriptions. In Britain, it has been found that most diagnoses coded in the general practice research database are valid [9]. However, many studies have pointed out that doctors tend to under-code [10–12] — not all of those diagnosed with an illness are coded as having that illness. Irish GPs have stated in the past that they often do not have the time to code patients [11] and previous research [13] has estimated that only 8% of patients in Irish general practice who had a mental disorder had been coded as having such. Therefore, we sought to use an electronic *finder* tool to encourage coding of SMDs inpatients.

Pharmacological treatment represents one of the primary means of mitigating severe mental illness symptoms. Certain anti-depressant, mood stabilising and anti-psychotic medications are thought to make a large contribution to obesity [2, 14]. This plays a significant role in the large health disparity between those with and without SMDs [2, 14]. There is also an association between anti-psychotics and diabetes [14, 15], and psychotropic drugs and cardiovascular disease risk [2, 14]. It is therefore important to understand the pharmacological treatment patterns of patients with SMDs in Ireland.

Consultation rates provide a proxy for the workload associated with groups of patients. Consultation rates for patients with a mental disorder are thought to be high in Irish general practice. O’Doherty et al. [16] estimated that patients with a mental disorder had a median of six consultations a year, compared to two consultations a year for patients without a mental disorder. It is therefore expected that consultation rates for patients with SMDs will be higher than a median of six per year.

Previous research has shown an association between socioeconomic status (SES) and SMD prevalence [17]. There is also an association between sex and SMD. Specifically, females are more likely than males to develop depression [18]. For schizophrenia and bipolar disorder, the relationship is less clear with studies finding mixed results [19, 20]. Gender [21, 22] and SES [17] also affect illness course and symptom expression in SMDs.

The objectives of the research were to establish:

- The prevalence of coding of SMDs in Irish general practice
- The pharmacological treatment of patients with SMD in Irish general practice
- The gender, age, age when first coded with a SMD, consultation rate and the medical card status of patients with SMD in Irish general practice

## Methods

Ethical approval for this study was obtained from the Irish College of General Practitioners Research Ethics Committee.

The study reported here had three major elements: (1) an electronic *finder* tool to assist GPs to find patients on their practice management software (PMS) system who have a SMD but have not been coded with one, (2) an electronic *register* tool to provide GPs with a list of patients on their PMS system who have been coded with a SMD and (3) an *uploader* to allow practices to send the relevant anonymous data to a central database. These elements formed part of a larger project examining the physical health of patients with a SMD.

### Finder

Patients with diagnosed illnesses are often not coded with these illnesses. Therefore, a *finder* tool was developed. The *finder* was developed by the researchers in consultation with two consultant psychiatrists and a GP with expertise in mental health. The purpose of the *finder* was to aid GPs in identifying and coding patients who have a SMD but have not been coded on their PMS with one. The *finder* provided a list of *active* adult patients who have not been coded with a SMD and are currently being prescribed any of the medications in Appendix Table 6 or had any of the following terms typed in the patient’s notes in the previous year: ‘schizophrenia’, ‘schizophrenic’, ‘bipolar’, ‘schizoaffective disorder’, ‘affective psychosis’ or ‘psychosis’. Prescriptions have been found to be the best indicator of depression in a general practice setting [10].

The list of patients provided by the *finder* to the GP were ordered from those who met the highest number of inclusion criteria to those who met the lowest number of inclusion criteria. The report provided the GP with details of each included patient’s name, age, sex, patient status, address, phone number, free text notes meeting inclusion criteria, medications prescribed and dosage of these medications. The GP then viewed the list and proceeded to code patients with the relevant SMD.

### Register

The *register* provided a list of patients in the GP’s practice who had been coded as having a SMD. The codes used to include patients in this register can be found in Appendix Table 5. The list of relevant medications and ICPC-2 and ICD-10 codes were decided upon by an expert group which included two consultant psychiatrists and a general practitioner.

### Uploader

The *uploader* sent anonymous information on patients coded with a SMD to a central database. This included their year of birth as well as all coded SMD diagnoses ever, and medicines prescribed, sex, patient status and number of consultations, for the 3 years prior to upload. With regard to coded SMD diagnoses, the data included all relevant ICD-10 and ICPC-2 diagnostic codes (Appendix Table 5). When a practice used the *uploader*, they were immediately sent an excel file with aggregated information on their practice’s patients, comparing this to all practices who uploaded data. The excel file contained information on the number of patients with SMD in the GP’s practice, as well as information related to their physical health such as smoking status, BMI levels and blood pressure. The excel file permitted the practices to undertake an audit of their practice against care guidelines and as undertaking audit is a requirement of the Medical Council in Ireland, this acted as an incentive for GP participation.

Patients coded with any of the codes in Appendix Table 5 were included in the *register* and *uploader*. The disease codes used to determine SMD diagnosis can be seen in Appendix Table 7.

### Practice recruitment

Three geographical areas — Dublin, Cork and Galway — were selected and expressions of interest were sought from GPs in these areas; 1,096 practices were contacted, 43 expressed an initial interest and 18 agreed to participate. At the time of writing, 11 practices had successfully uploaded data from two practice software systems which implemented the tools. These two systems service approximately 65–70% of GP practices nationally. Consultation figures from one of the PMS systems were excluded from the analysis, due to difficulties experienced by GPs in recording consultations. This led to data from this system being excluded from the prescriptions analysis because it was not possible to establish whether a patient had had a consultation in the preceding year.

### Data/Definitions

The age figure was calculated based on year of birth as it was not possible to access exact date of birth because this was considered patient identifiable information.

Descriptive statistics are provided on patient status; there are three types of patient status: ‘private patients’, ‘public patients’ and ‘doctor visit card patients’. Public patients are those who are entitled to a medical card, which is a means tested scheme. The medical card provides free access to certain medical and surgical services in Ireland, including free GP care [23]. The doctor visit card entitles the holder to visit the GP for free [23]. The income thresholds vary depending on age, marital status and having children [23]. All carers along with everyone over the age of 70 years or under the age of 6 years are entitled to a doctor visit card [22]. All patients who do not have a medical card or doctor visit card are classified as private patients. Private patients pay for GP services and are not required to register with one GP.

Patient status is used in this paper as a proxy for SES. Public patients represent the most deprived in society. Doctor visit card patients represent those on a higher income than public patients, and private patients represent those on a higher income than both public patients and doctor visit card patients.

The size of the practice population was established by the GP conducting an audit of their respective system to establish the number of *active* patients aged 18 or over registered in the practice. All patients who are registered or visit the practice and therefore entered on the PMS system are by default classified as *active*; in order for their status to differ from this, they must be actively reclassified as inactive, deceased or archived.

For age when first coded with a SMD, patients coded after the GP was instructed to use the *finder* tool were excluded. This step was applied due to likelihood that these patients had been diagnosed with a SMD at an earlier point but had not been coded.

If a patient was found to have more than one consultation in a single day, then this was considered one single consultation on that day. This was done because many patients appeared to be having several consultations on a single day but this was most likely caused by an error, by having a consultation with the GP and with the nurse or due to double visits been booked for patients who might require a longer visit.

The number of GPs is quantified by the method of whole time equivalent (WTE), whereby a full-time GP is counted as one WTE GP while a part-time GP is counted as half WTE GP.

## Results

Eleven practices provided data for the study. The mean number of full-time GPs was 2.3 (range = 1–4), and the mean number of part-time GPs was 1.3 (range = 0–7). The total number of WTE GPs was 35 with an average WTE of 3.2 GPs per practice.

The overall practice population across all practices was 80,543. A total of 2,337 patients had been coded with a SMD, representing 2.9% of the practice populations. Most of these patients were coded with (recurrent) depressive disorder; 2.4% (*n* = 1,964) of the practice populations. The mean age of patients at the time of upload was 53.8 years (range = 18–103 years) (Table 1). The median consultation rate in the previous 3 years for patients with a SMD was 20.

**Table 1:** Patients coded with SMDs, average age when first coded with the illness and median number of consultations for each SMD

Disorder	Percentage of practice population ever coded with SMD (95% CI)	Age at time of upload Mean (*SD*)	Age when first coded Mean (*SD*)	Number of consultations in the last 3 years Median (*Interquartile range*)
(Recurrent) depressive disorder	2.44% (2.33 to 2.55%)	53.92 (*18*.*15*)	45.82 (*17.42*)	19.5 (*30*)
Schizophrenia	0.29% (0.25 to 0.33%)	53.48 (*15*.*14*)	45.25 (*14.50*)	24.5 (*34*)
Bipolar disorder	0.26% (0.23 to 0.30%)	53.29 (*16.09*)	46.71 (*16.22*)	22 (*37*)

After instructing the GPs to use the finder, an additional 3.8% (*n* = 74) were coded with (recurrent) depressive disorder, followed by 11.2% (*n* = 26) coded with schizophrenia and 28.2% (*n* = 59) coded with bipolar disorder.

Amongst patients who have been coded as having (recurrent) depressive disorder, 49.9% (*n* = 977) were private patients, whereas 22.9% (*n* = 53) of those coded with schizophrenia were private patients (Table 2).

**Table 2:** Patients’ status for each SMD

Disorder	Private patient (95% CI)	GMS/doctor visit card patient (95% CI)
(Recurrent) depressive disorder	49.9% (47.7 to 52.1%)	50.1% (47.9 to 52.3%)
Schizophrenia	22.9% (18.0 to 28.8%)	77.1% (71.2 to 82.0%)
Bipolar disorder	38.8% (32.4 to 45.5%)	61.2% (54.5 to 67.6%)

Of the patients who were coded with a SMD, 97.2% (*n* = 2,272) were coded with a single SMD only, 2.6% (*n* = 61) were coded with two SMDs and 0.2% (*n* = 4) patients were coded with all three SMDs. Of those who have a dual diagnosis, 37.7% (*n* = 23) were coded with depression and schizophrenia, 49.2% (*n* = 30) were coded with bipolar disorder and depression and 13.1% (*n* = 8) of patients were coded with schizophrenia and bipolar disorder. For those coded with a SMD, 64.8% (*n* = 1,515) were female (Table 3).

**Table 3:** Sex of patients for each SMD

Disorder	Female (95% CI)	Male (95% CI)
(Recurrent) depressive disorder	68.0% (65.9 to 70.1%)	32.0% (29.9 to 34.1%)
Schizophrenia	42.5% (36.2 to 48.8%)	57.5% (51.2 to 63.8%)
Bipolar disorder	57.4% (50.6 to 63.9%)	42.6% (36.1 to 49.4%)

For (recurrent) depressive disorder, the highest prescription rate was for anti-depressants; 57.9% (*n* = 710) of patients coded with (recurrent) depressive disorder who had visited in the preceding year were prescribed at least one anti-depressant medication in the preceding year. For schizophrenia, 64.0% (*n* = 103) were prescribed at least one anti-psychotic medication and for bipolar disorder 75.2% (*n* = 94) were prescribed at least one anti-psychotic medication (Table 4).

**Table 4:** Patients with one SMD diagnosis only (who have visited in the preceding year) prescribed at least one medication in the previous year for each SMD

Disorder	Anti-psychotic (ATC code: N05A)	Anti-depressant (ATC code: N06A)	Anxiolytic (ATC code: N05B)	Hypnotics & sedatives (ATC Code: N05C)
(Recurrent) depressive disorder	12.9% (*n* = 158)	57.9% (*n* = 710)	18.2% (*n* = 223)	20.5% (*n* = 252)
Schizophrenia	64.0% (*n* = 103)	18.2% (*n* = 26)	18.2% (*n* = 26)	20.3% (*n* = 29)
Bipolar disorder	75.2% (*n* = 94)	29.6% (*n* = 37)	33.2% (*n* = 29)	34.0% (*n* = 30)

## Discussion

This study has revealed that electronic tools to assist GPs in finding people with mental health diagnoses in GP software systems can greatly increase coding of SMDs; this was particularly the case for schizophrenia and bipolar disorder where 11% and 28% respectively of those coded were coded after the finder tool was used. This study also provides further evidence of high consultation rates amongst people with mental health conditions: The median number of consultations in the last 3 years for patients with (recurrent) depressive disorder was 19.5, whereas for patients with schizophrenia it was 24.5, and for patients with bipolar disorder, it was 22. Previous research [16] in Irish general practice has found that patients with a documented mental health condition had had a median of six consultations in the previous year, and those without a mental health condition had a median of two in the previous year. The average age when first coded was similar for all three SMDs: 45.8 years for (recurrent) depressive disorder, 45.3 years for schizophrenia and 46.7 years for bipolar disorder.

Hasin et al. [24], using self-reported survey, estimated the lifetime prevalence of major depressive disorder in the United States adult population at 13.23%, far greater than the 2.4% figure we found for (recurrent) depressive disorder.

A systematic review [25] estimated the prevalence of bipolar disorder in primary care internationally between 0.5 and 4.3% of practice populations. Structured interviews with patients or screening questionnaires were used to establish prevalence. A study of prevalence of bipolar disorder in one area in Ireland (County Monaghan) estimated a lifetime prevalence of 0.4% [26], greater than the 0.26% found in this study.

Estimated schizophrenia prevalence in Ireland is 0.4% [27]. A systematic review [28] estimated lifetime schizophrenia prevalence at 0.4%, similar to the 0.3% found in this study.

Given the percentage of patients coded after GPs were provided with the *finder* tool was 4% for (recurrent) depressive disorder, 11% for schizophrenia and 28% for bipolar disorder, it seems likely that there are many patients with a SMD who have not been coded with SMD. This is reinforced by the disparities in prevalence rates when compared to previous research outlined above. However, these results undermine the estimate that only 8% of general practice patients in Ireland with any mental disorder were coded with that disorder [13].

The main medication patients with schizophrenia and bipolar disorder were prescribed was anti-psychotics with 64% and 75% of patients respectively being prescribed at least one anti-psychotic in the preceding year. A study of Irish primary care patients found that 78% of patients who had experienced psychosis were treated with anti-psychotic medication [13]. For (recurrent) depressive disorder, anti-depressants were the most prescribed medication; 58% of patients had at least one anti-depressant prescription in the preceding year. In the previously mentioned study of Irish primary care patients, 74% of patients with depression were treated with anti-depressants [13]. The prescription rates found in the present study appear to be low given that these are the main pharmacological treatments for the respective conditions [29, 30]. For patients with (recurrent) depressive disorder, it may be the case that many are engaging in counselling or psychotherapy instead of medication. For patients with schizophrenia, it may be the case that some patients are getting their medication via injection from the mental health clinic or from the practice nurse and therefore, it is not read as a prescription on the PMS.

With regard to SES, as of April 2018, 30.2% (*n* = 1,103,937) of the Irish adult population (aged 18 and over) were eligible to be public patients. Also, 5.0% (*n* = 183,935) were GP visit card eligible [31]. The denominator used is the 2018 census figure which shows that there are 3,657,089 people aged 18 and over in Ireland [32]. Therefore, for all three diagnoses, if a patient is a public patient, they are more likely than a private patient to have been coded with a SMD. The parallel between patient’s status and being diagnosed with a SMD is clearest in patients with schizophrenia where 74% of patients coded with schizophrenia are medical card holders. The previous studies also found a negative correlation between SES and prevalence of mental illness [17]. However, this correlation may be confounded by attendance rates; public patients or doctor visit card patients are not charged for attending the GP, and private patients are charged and have lower consultation rates than public patients [33]. Therefore, it could be that public/doctor visit card patients are more likely to be identified and diagnosed as having a SMD.

Over two-thirds (68%) of those coded with (recurrent) depressive disorder were female. This is in line with previous research [18] which has found that almost twice as many women than men have been diagnosed with depression. There are several possible explanations for this disparity. Health-seeking behaviour has been found to be different [34] between males and females. Also, gender-based violence and SES are thought to contribute to the disparity [35]. It is thought that there is no difference in prevalence between males and females in terms of bipolar disorder [19]. In this study, the disparities between genders are less prominent for bipolar disorder. For schizophrenia, it is unclear whether gender differences exist [19, 20], with some studies suggesting a higher prevalence amongst men [20]. This study found that men were more likely to be coded with schizophrenia.

In terms of the low coding figures, doctors tend not to code for several reasons including lack of time [4] and concern that coding will negatively affect patient consultations [4]. Also, due to the stigma associated with mental illness [36], doctors may be disinclined to code patients with a SMD. Another reason why patients might not be coded with a SMD is lack of diagnosis; the accuracy of GPs’ mental health diagnoses is questioned by some studies [37, 38].

There are several study limitations in relation to the SMD coding figures reported. Firstly, upon viewing the list of patients the *register* provided, some GPs removed patients with SMDs who were deceased or no longer in the practice. However, they were not carrying out the same process for the overall practice population. Also, it was only suggested and not enforced that GPs use the *finder* and *register* tools before uploading data. Another reason is that a patient with a SMD may not appear in the *finder* tool; it is possible that not all patients with SMDs had been prescribed a relevant medication or had a relevant word entered into their notes.

The fact that there is no code for bipolar disorder in ICPC-2, which is the main coding system used by GPs in this study, may contribute to the low levels of coding for bipolar disorder. Also, the diagnosis of recurrent depressive disorder is not clear from the available codes in ICPC-2. In ICPC-2, the only relevant options are P76 (*Depression*) and P03 (*Feeling depressed*). This study included P76 which is not necessarily recurrent and may only include a single episode. Also, P73 affective psychosis was included as a code for bipolar disorder as bipolar disorder is a sub-code of P73 affective psychosis; however, it might not be the case that all patients coded with P73 have bipolar disorder.

It is also worth noting that there is a selection bias; participating GPs self-selected and therefore it is possible that they have a special interest in the topic and therefore might be more likely than other GPs to recognise, diagnose and code SMDs. However, looking at the practice population/GP ratio and the number of WTE GPs per practice, the profile of the participating practices is similar to that reported nationally [39, 40]. Medical card/GP visit card is a limited proxy for socioeconomic status, as it is binary and has higher income thresholds for older adults and there are several exceptions for medical card provision which are not clear proxies for socioeconomic status.

An understanding of coding, consultations and treatment of SMDs in Ireland can help inform the resourcing of general practice and interventions for patients with SMDs. Improving the validity of diagnostic coding should be a priority in Ireland in order to provide more accurate prevalence and impact data. The British quality and outcomes framework gave financial incentives for disease coding which led to a large increase in the coding of chronic disease [41–43]. It is acknowledged that the new chronic disease contract introduced in January 2020 in Ireland on a phased basis may assist in this regard. Future interventions for patients with SMDs should take into account the disproportionate number of female patients and public patients amongst those with a SMD. Future research might seek to establish the exact mechanisms through which gender and SES affect SMD incidence.

### Electronic supplementary material

Below is the link to the electronic supplementary material.

Supplementary file1 (DOCX 29 KB)

Supplementary file2 (DOCX 29 KB)

Supplementary file3 (DOCX 29 KB)

Supplementary file4 (DOCX 29 KB)

Supplementary file5 (DOCX 29 KB)

## Data Availability

Data are available on reasonable request to the corresponding author.

## Appendix

**Table 5:** Disease code inclusion criteria for register and uploader

ICPC-2	Clinical diagnosis	ICD-10	Clinical diagnosis
P71	Organic psychosis other	F09	Unspecified organic or symptomatic mental disorder
P72	Schizophrenia	F20	Schizophrenia
P73	Affective psychosis	F25	Schizoaffective disorder
P76	Depressive disorder	F29	Unspecified nonorganic psychosis
P98	Psychosis NOS/other	F30	Manic episode
		F31	Bipolar affective disorder
		F33	Recurrent depressive disorder
		F39	Unspecified mood [affective] disorder

**Table 6:** Medication inclusion criteria for finder, register, and uploader

Antipsychotic Medicines		Cholinesterase Inhibitors		Other anti-dementia drugs	
**ATC**	**Generic name**	**ATC**	**Generic name**	**ATC**	**Generic name**
N05AH04	Quetiapine	N05B	Anxiolytics – Any Anxiolytic with ATC CODE starting with “N05B”	N06DX01	Memantine
N05AD01	Haloperidol				
N05AB06	Trifluoperazine				
N05AX08	Risperidone	N05C	Hypnotics and sedatives – Any Hypnotics and sedative with ATC CODE starting with “N05C”		
N05AH03	Olanzapine				
N05AB02	Fluphenazine				
N05AX13	Paliperidone				
N05AH02	Clozapine	N06B	Psychostimulants, Agents used for ADHD and Nootropics – Any Psychostimulants, Agents used for ADHD and Nootropics with ATC CODE starting with “N06B”		
N05AA01	Chlorpromazine				
N05ALO5	Amisulpride				
N05AX12	Aripiprazole				
N05ALO1	Sulpiride				
N05AF05	Zuclopenthixol				
N05AD07	Benperidol	N06C	Any Psycholeptics & Psychoanaleptics in combination with ATC CODE starting with “N06C”		
N05AF01	Flupentixol				
N05AA03	Promazine				
N05AC04	Pipothiazine				
N05AE03	Sertindole				
N05AE04	Ziprasidone				
N05AE05	Lurasidone				
N05AG02	Pimozide				
N05AH05	Asenapine				
N05AX13	Paliperidone				
N05AN01	Lithium 24				

**Table 7:** The disease codes used to determine SMD diagnosis

Depression – P76, F33
Schizophrenia – P72, F20, F25
Bipolar Disorder – F25, F33, P73

## References

[1] Newcomer JW, Hennekens CH (2007). Severe mental illness and risk of cardiovascular disease. JAMA.

[2] De Hert M, Correll CU, Bobes J et al (2011) Physical illness in patients with severe mental disorders. I. Prevalence, impact of medications and disparities in health care. World Psychiatry 10(1):52–77 10.1002/j.2051-5545.2011.tb00014.x PMC3048500 21379357

[3] Whiteford HA, Degenhardt L, Rehm J (2013). Global burden of disease attributable to mental and substance use disorders: findings from the Global Burden of Disease Study 2010. The Lancet.

[4] Chesney E, Goodwin GM, Fazel S (2014). Risks of all-cause and suicide mortality in mental disorders: a meta-review. World Psychiatry.

[5] Whitford DL, Copty M (2005). General practice in Ireland: are we equipped to manage mental health. Irish Journal of Psychological Medicine.

[6] Copty M, Whitford DL (2005). Mental health in general practice: assessment of current state and future needs. Irish Journal of Psychological Medicine.

[7] Department of Health and Children (2006) A vision for change: Report of the expert group on mental health policy. Dublin: Government Publications Office.https://www.hse.ie/eng/services/publications/mentalhealth/mental-health---a-vision-for-change.pdf. Accessed 18 September 2020

[8] Department of Health (2020) Sharing the vision: a mental health policy for everyone. gov.ie - Sharing the Vision: A Mental Health Policy for Everyone (www.gov.ie). Accessed 7 July 2021.

[9] Khan NF, Harrison SE, Rose PW (2010). Validity of diagnostic coding within the General Practice Research Database: a systematic review. Br J Gen Pract.

[10] Joling KJ, van Marwijk HW, Piek E, wt al.  (2011). Do GPs’ medical records demonstrate a good recognition of depression? A new perspective on case extraction. J Affect Disord.

[11] Kennedy C, O’Brien C, Collins C (2014) Experience of diagnostic coding in Irish general practice: the practice perspective. JMED Research Article ID. 10(2014.583528).

[12] Swan D, Hannigan A, Higgins S et al (2017) Development and implementation of a ‘mental health finder’software tool within an electronic medical record system. Ir Med Sci (1971). 186(1):191–200 10.1007/s11845-016-1541-4 28050808

[13] Gleeson M, Hannigan A, Jamali R, er al.  (2016). Using electronic medical records to determine prevalence and treatment of mental disorders in primary care: a database study. Irish Journal of Psychological Medicine.

[14] American Diabetes Association (2004). Consensus development conference on antipsychotic drugs and obesity and diabetes. Diabetes Care.

[15] Reynolds GP, Kirk SL (2010). Metabolic side effects of antipsychotic drug treatment – pharmacological mechanisms. Pharmacol Ther.

[16] O’Doherty J, Hannigan A, Hickey L et al (2018) The prevalence and treatment of mental health conditions documented in general practice in Ireland. Ir J Psychol Med pp.1–8 10.1017/ipm.2018.48 30486911

[17] Hudson CG (2005). Socioeconomic status and mental illness: tests of the social causation and selection hypotheses. Am J Orthopsychiatry.

[18] Munce SE, Stewart DE (2007). Gender differences in depression and chronic pain conditions in a national epidemiologic survey. Psychosomatics.

[19] Merikangas KR, Akiskal HS, Angst J et al (2007) Lifetime and 12-month prevalence of bipolar spectrum disorder in the National Comorbidity Survey replication. Arch Gen Psychiatry 64(5):543–52 10.1001/archpsyc.64.5.543 PMC1931566 17485606

[20] Ochoa S, Usall J, Cobo J et al (2012) Gender differences in schizophrenia and first-episode psychosis: a comprehensive literature review. Schizophrenia Res Treat 2012 10.1155/2012/916198 PMC3420456 22966451

[21] Hendrick V, Altshuler LL, Gitlin MJ (2000). Gender and bipolar illness. J Clin Psychiatry.

[22] Moriarty PJ, Lieber D, Bennett A (2001). Gender differences in poor outcome patients with lifelong schizophrenia. Schizophr Bull.

[23] Citizens Information (2020) Medical cards and GP visit cards. http://www.citizensinformation.ie/en/health/medical_cards_and_gp_visit_cards/. Accessed 18 September 2020.

[24] Hasin DS, Goodwin RD, Stinson FS (2005). Epidemiology of major depressive disorder: results from the National Epidemiologic Survey on Alcoholism and Related Conditions. Arch Gen Psychiatry.

[25] Cerimele JM, Chwastiak LA, Dodson S (2014). The prevalence of bipolar disorder in general primary care samples: a systematic review. Gen Hosp Psychiatry.

[26] Scully PJ, Owens JM, Kinsella A (2004). Schizophrenia, schizoaffective and bipolar disorder within an epidemiologically complete, homogeneous population in rural Ireland: small area variation in rate. Schizophr Res.

[27] Nuallain MN, O'Hare A, Walsh D (1990) The prevalence of schizophrenia in three counties in Ireland. Acta Psychiatry Scand 82(2):136–40 10.1111/j.1600-0447.1990.tb01370.x 2239357

[28] Saha S, Chant D, Welham et al (2005) A systematic review of the prevalence of schizophrenia. PLoS medicine 2(5), p.e141 10.1371/journal.pmed.0020141 PMC1140952 15916472

[29] Malhi GS, Adams D, Berk M (2010). The pharmacological treatment of bipolar disorder in primary care. Med J Aust.

[30] Mayo Clinic Staff (2018) Schizophrenia diagnosis & treatment. https://www.mayoclinic.org/diseases-conditions/schizophrenia/diagnosis-treatment/drc-20354449. Accessed 18 September 2020.

[31] Health Services Executive (2019) Primary care reimbursement. Service https://www.hse.ie/eng/staff/pcrs/. Accessed 22 January 2019

[32] Central Statistics Office (2019) Population estimates from 1926 by single year of age, sex and year. https://www.cso.ie/px/pxeirestat/Statire/SelectVarVal/Define.asp?maintable=PEA11&PLanguage=0. Accessed 22 January 2019

[33] Behan W, Molony D, Beame C (2013). (2013) Are Irish adult general practice consultation rates as low as official records suggest? A cross sectional study at six general practices. Ir Med J.

[34] Carretero MT, Calderón-Larrañaga A, Poblador-Plou B, ety al.  (2014). Primary health care use from the perspective of gender and morbidity burden. BMC Womens Health.

[35] Astbury J (1999) Gender and mental health. Harvard center for population and development studies. https://www.hsph.harvard.edu/population-development/. Accessed 18 September 2020

[36] Corrigan PW, Watson AC (2002) Understanding the impact of stigma on people with mental illness. World Psychiat 1(1):16 PMC1489832 16946807

[37] Das AK, Olfson M, Gameroff MJ (2005). Screening for bipolar disorder in a primary care practice. JAMA.

[38] Mitchell AJ, Vaze A, Rao S (2009) Clinical diagnosis of depression in primary care: a meta-analysis. The Lancet 22:374(9690):609–19 10.1016/S0140-6736(09)60879-5 19640579

[39] Smith S, Walsh B, Wren M et al (2019) Geographic profile of healthcare needs and non-acute healthcare supply in Ireland. ESRI Research Series: 90. Dublin: Eco Social Res Ins 2019. Available from: https://www.esri.ie/pubs/RS90_0.pdf. Accessed 30th June 2021

[40] Collins C, Homeniuk R (2020) How many general practice consultations occur in Ireland annually? Cross-sectional data from a survey of general practices. BMC Fam Pract 22(40). 10.1186/s12875-021-01377-0 PMC7896162 33610171

[41] Simpson CR, Hannaford PC, Lefevre K (2006). Effect of the UK incentive-based contract on the management of patients with stroke in primary care. Stroke.

[42] McGovern MP, Williams DJ, Hannaford PC (2008). Introduction of a new incentive and target-based contract for family physicians in the UK: good for older patients with diabetes but less good for women?. Diabet Med.

[43] McGovern MP, Boroujerdi MA, Taylor MW (2008). The effect of the UK incentive-based contract on the management of patients with coronary heart disease in primary care. Fam Pract.

